# Covert Tracking: A Combined ERP and Fixational Eye Movement Study

**DOI:** 10.1371/journal.pone.0038479

**Published:** 2012-06-13

**Authors:** Alexis D. J. Makin, Ellen Poliakoff, Rochelle Ackerley, Wael El-Deredy

**Affiliations:** 1 School of Psychological Sciences, University of Manchester, Manchester, United Kingdom; 2 Department of Experimental Psychology, University of Liverpool, Liverpool, United Kingdom; 3 Faculty of Life Sciences, University of Manchester, Manchester, United Kingdom; 4 Sahlgrenska Academy, University of Gothenburg, Gothenburg, Sweden; University of Muenster, Germany

## Abstract

Attention can be directed to particular spatial locations, or to objects that appear at anticipated points in time. While most work has focused on spatial or temporal attention in isolation, we investigated covert tracking of smoothly moving objects, which requires continuous coordination of both. We tested two propositions about the neural and cognitive basis of this operation: first that covert tracking is a right hemisphere function, and second that pre-motor components of the oculomotor system are responsible for driving covert spatial attention during tracking. We simultaneously recorded event related potentials (ERPs) and eye position while participants covertly tracked dots that moved leftward or rightward at 12 or 20°/s. ERPs were sensitive to the direction of target motion. Topographic development in the leftward motion was a mirror image of the rightward motion, suggesting that both hemispheres contribute equally to covert tracking. Small shifts in eye position were also lateralized according to the direction of target motion, implying covert activation of the oculomotor system. The data addresses two outstanding questions about the nature of visuospatial tracking. First, covert tracking is reliant upon a symmetrical frontoparietal attentional system, rather than being right lateralized. Second, this same system controls both pursuit eye movements and covert tracking.

## Introduction

Selective attention enhances sensory inputs that are relevant to current goals, and inhibits task-irrelevant inputs. A great deal of research has been carried out into how people can shift their attention to spatial locations *covertly*, that is, without moving their eyes [1]. The neural correlates of this have been examined using Event Related Potentials (ERPs), revealing that the P1 potential (generated by the extrastriate visual cortex at 100–130 ms post-stimulus), is larger when a stimulus is presented in an attended location and reduced for unattended locations [2]. More recently, researchers have considered attention to stimuli that appear at an expected point in *time*: Doherty et al. [3] presented a single dot target that moved rightwards in a series of discrete steps, before disappearing behind an occluder. ERPs were recorded at the point when the target reappeared after occlusion. The P1 component was largest when the target reappeared at the expected time and expected location, demonstrating that selective attention can operate in both the spatial and temporal domains [4].

Rather than examining the discrete shifts in covert attention, which have been the topic of most literature to date, here we explore what happens when people pay attention to smoothly moving objects. In this case, it is necessary to continuously attend to the correct *location* at the correct *time*, so attention must be coordinated across the spatial and temporal domains. This kind of spatiotemporal coordination is a phylogenetically recent development, which is carried out by specialized brain systems [5] and is essential for many human activities, such as driving [6] and playing sports [7]. As well as attending to visible moving objects, people are also able to attend to a moving object that is occluded for a short period of time [8], [9]. Participants are faster to respond to stimuli presented in the current location of the occluded target, suggesting that the ‘spotlight’ of spatial attention continuously follows the invisible motion [10]. Occluded tracking is of particular interest since it allows us to isolate the relative contribution of bottom-up sensory inputs and top-down predictive mechanisms. When attending to or attempting to track a visible moving target, bottom-up sensory information about target velocity and top-down predictive mechanisms are employed. In contrast, predictive signals based on the remembered velocity, must operate alone once occlusion is cortically registered at around 100–200 ms after occlusion onset [11], [12].

We aimed to answer two important questions about covert tracking. First, whether right hemisphere regions predominantly mediate attentive tracking. Mesulam [13] described a network of brain regions that are crucially involved in spatial attention, centered on the posterior parietal cortex and the frontal eye fields (FEFs). Mesulam further suggested that while the left frontoparietal system is involved in shifting attention to the contralateral (right) hemifield, the right frontoparietal system is involved in directing attention to *both* contralateral (left) *and* ipsilatateral (right) hemifields. This conclusion is supported by neuropsychological studies, which consistently find that hemispatial neglect is more profound after damage to the right parietal lobe [14]. However, functional magnetic resonance imaging (fMRI) findings have not universally supported a right-hemisphere model of attentive tracking. In general, researchers have found that the frontoparietal system is activated *bilaterally* during attentive tracking, with some inconsistent lateralization depending on specific eye movement instructions or memory requirements [15]–[20].

These fMRI studies usually measured brain activity in blocks involving equal presentations of leftward and rightward motion. They do *not* imply that the amount of activity in the left or right frontoparietal system is independent of the current focus of spatial attention; but neither do they strongly suggest any special status for the right hemisphere. Nevertheless, the poor temporal resolution of fMRI may mask lateralized activations that occur at particular periods within a trial. In the current work, we took advantage of the high temporal resolution of EEG to explore hemispheric asymmetry during covert tracking.

In our previous work, participants covertly tracked rightward motion, and we recorded a right-lateralized ERP that peaked after the target crossed the midline [21]. However, it is not certain whether this was due to the rightward direction of covert tracking, or cerebral asymmetry. In the current work, therefore, we compared moving targets that travelled leftwards or rightwards. If topographic development of ERPs in the leftward condition is not a mirror image of the rightward condition, then we can conclude that covert tracking depends on cortical networks that are also asymmetric. Another possibility is that *visible tracking* is bilateral (with relative activity in the each hemisphere depending on the current focus of spatial attention), but *occluded tracking* recruits additional right hemisphere networks (independent of the current focus of spatial attention). This is suggested by some neuroimaging studies, in which additional right hemisphere activations are evident during occlusion [17], [20]. Therefore, we might find symmetrical ERP development in during tracking of visible, but not occluded targets. Another advantage of comparing visible and occluded tracking in our previous work was that it allowed us to record the change in brain activity corresponding to the onset of memory-guided tracking [21], and we expect a similar Occlusion Related ERP here.

The second question is about the role of the oculomotor system in covert tracking. The *pre-motor theory of attention* [22] suggests that covert shifts of spatial attention are guided by the pre-motor mechanisms responsible for saccadic eye movements, even when eye movements are never executed [23], [24]. Inspired by this theory, we hypothesized that the neural mechanisms involved in smooth pursuit eye movements to follow moving targets also mediate covert tracking during fixation [18], [21], [25]. Neuroimaging studies have provided strong support for this idea, by demonstrating that covert attentive tracking activates brain regions such as the middle temporal area, the intra-parietal sulcus and the FEFs, which are known to control eye movements [15].

We tested the involvement of the oculomotor system during covert tracking, by recording small changes in eye position, which always occur during fixation (fixational eye movements; see [26], for a review). Previously, we found that average eye position shifted rightwards by about 0.1° when participants covertly tracked rightward moving targets, even on trials where participants maintained fixation throughout [25]. These fixational eye movements are thought to reflect covert oculomotor activation, and may index the orientation of spatial attention [27], [28], although fixational eye movements have other functions as well [29]. The current study allowed us to compare ERPs and fixational eye movements on the same participants, carrying out the same tasks. Most importantly, this within-subjects design allowed us to compare the precise temporal development of ERPs and fixational eye movements. If these measures were closely related in time, it would suggest that they reflect the same underlying neurocognitive operations.

We presented leftward or rightward moving dot targets, which either remained visible throughout (Visible Task) or were occluded for at least 500 ms mid trajectory (Occluded Task). Targets moved at 12 or 20°/s across a central fixation point, where participants held their gaze throughout the trial. The Visible and Occluded Tasks were presented in separate blocks, and participants were given behavioral tasks designed to encourage covert attentive tracking. The rightward conditions of this experiment were similar to those reported by Makin et al. [21]. However, in the current work, we simultaneously measured the development of ERPs in the leftward and rightward conditions with scalp electrodes, and the direction of fixational eye movements with a desk-mounted eye tracker.

## Material and Methods

### Ethics Statement

The protocol for this study was approved by the School of Psychological Sciences Research Ethics Committee at the University of Manchester (reference 306/05) and in accordance with the Declaration of Helsinki. Written informed consent was obtained from all participants.

### Participants

Twenty University of Manchester students with normal or corrected-to-normal vision (4 male, aged 18–29, all right handed) took part in the study and received £20 or course credit as an incentive. Two participants were excluded from all analysis because their electroencephalogram (EEG) data was unsuitable (see section 2.6).

### Apparatus

Participants sat at a table in a dimly lit room and were positioned 75 cm from a 30×40 cm CRT monitor that subtended approximately 30° of their visual field. Visual stimuli were presented using a VISAGE Visual Stimulus Generator (Cambridge Research Systems, Rochester, UK). During EEG recording, the participant’s head was stabilized with a chin rest and they placed their left and right index fingers respectively on the ‘A’ and ‘L’ buttons of a computer keyboard, which they used to enter their responses.

EEG recording and analysis followed Makin et al. [21]. Continuous EEG was recorded using Synamps (Neuroscan Inc., Charlotte, NC) from 61 AgCl scalp electrodes (position according to the extended 10–20 system) relative to a CZ reference, and subsequently average-referenced offline. Vertical and horizontal electro-oculograms were recorded with separate electrodes placed above and below the left eye and on the outer canthi of both eyes. Impedance was kept below 5 KΩ throughout and EEG was sampled at 500 HZ. Bandpass filters were set at 0.01 Hz –100 Hz.

Eye position was sampled at 50 Hz with a remote Eye Trac 6000 system (ASL, Bedford, MA) infrared eye tracking system. The eye tracker was mounted on the table between the participant and the stimulus monitor. Calibration involved asking participants to look at each of 9 points spaced evenly around the target trajectory. Calibration was conducted before the experiment and between experimental blocks.

### Visible Task Procedure

In the Visible Task, moving dot targets were presented 60 times in each of four conditions, [Speed (12, 20°/s)×Direction (leftward, rightward motion)]. The experiment was divided into 6 blocks with 10 repeats of each condition per block. The trials in each block were presented in a pseudo-random order, with no more than 3 repeats of a single condition presented sequentially. Each block contained an additional 8 oddballs (16.7%), which included an unexpected change in velocity, giving 48 oddballs in total. Half the participants completed the blocks in reverse order. During each trial, the participants were required to fixate their gaze on a central cross, and to look out for the rare velocity change oddball trials.

**Figure 1:**
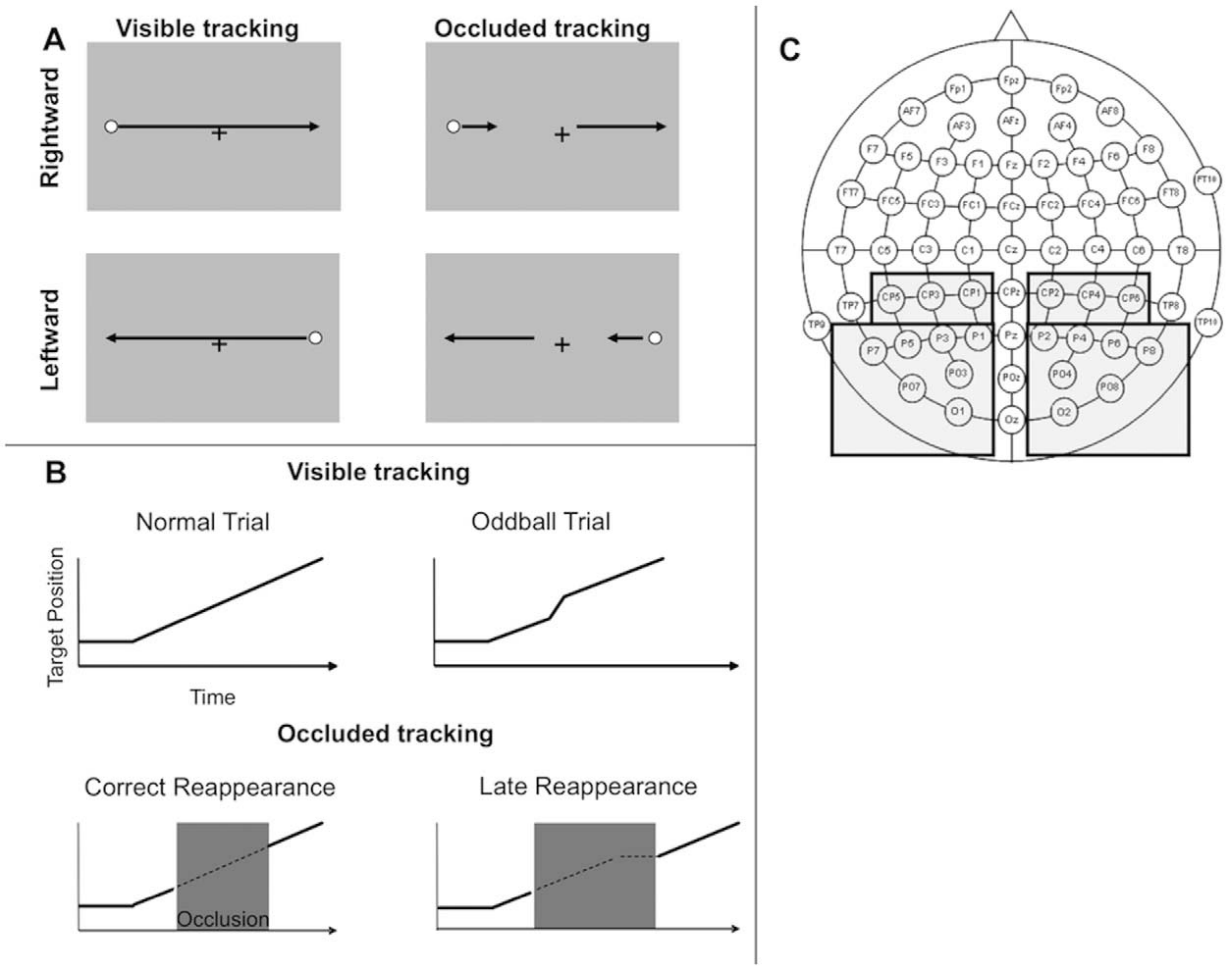
The Experimental Tasks and Set-up. A) Diagram of the basic tasks. B) Upper panels: Schematic of the target position vs. time in the Visible Task. Lower panels: schematic of target position vs. time in the Occluded Tracking Task. C) The layout of scalp electrodes. Grey shaded regions show the left and right clusters that were used for all analysis.

In rightward trials, the target remained static 1.8° from the left hand edge of the screen for 600 ms. This static period alerted the participants that the trial was about to start and prevented evoked potentials produced by visual onset from overlapping with motion related brain activity. The target then moved rightward for 26.25°, with a path centered horizontally on the fixation point. The vertical position of the fixation point was 5° above the screen center, at approximate eye level, and the vertical position of target path was slightly above the fixation cross (by half the diameter of the dot target, 0.22°). Motion duration was 1312 ms in the 20°/s conditions, and 2187 ms in the 12°/s condition. The velocity change oddball, during which the target velocity doubled in speed for 100 ms, could occur at any point, selected at random, during the central 17.5° of the target’s path. This corresponded to durations of 875 ms in the 20°/s conditions and 1458 ms in the 12°/s conditions. After the target reached the end of its trajectory, there was a 300 ms pause and the response screen appeared. The leftward trials were a mirror image of the rightward trials (Figure 1A, B).

The response screen consisted of two words, presented to the left and right of the fixation cross: ‘NORMAL’ and ‘ODDBALL’. Participants made an unspeeded response, with the position of the words indicating which button corresponded to normal and oddball trials. The hand used to report the different judgments varied trial-by-trial and was balanced across conditions. This design prevented asymmetric motor response preparation during the visual motion [30]. There was then a 1.2 second pause before the next trial, during which the participant fixated.

Before the experiment, a practice block of 16 trials was presented. The practice block included 4 oddballs (1 repeat of each condition). The remaining 12 trials were normal (3 repeats of each condition) with equal number of left and right hand responses.

### Occluded Task Procedure

In the Occluded Task, moving dot targets were presented 60 times in each of four conditions: [(12, 20°/s)×(leftward, motion)]. As with the Visible Tracking Task, the target remained static 1.8° from either the edge of the screen for 600 ms and then moved leftwards or rightwards for 26.25° (Figure 1A, B). The target’s path was centered on the fixation point. The first 5.95° of target motion was visible, corresponding to durations of 300 ms in the 20°/s condition and 500 ms in the 12°/s condition. The target then disappeared from sight behind an invisible occluder (a rectangle of the same color as the background). There were 5 different occluder sizes, ranging from 10.21 to 13.71° in 0.875° increments, which produced occlusion duration times of 850–1142 ms for the on-time 12°/s targets and 510–685 ms for the on-time 20°/s targets. For late reappearance trials, the target reappeared from behind the occluder in the same positions as the on-time reappearance trials, but 300 ms too late. After reappearance, the target travelled to the end of its path, so, motion duration for on-time trials was identical to that of the normal Visible Task trials. There was then a 300 ms pause before the response screen appeared. The leftward trials were a mirror image of the rightward trials (Figure 1A, B). Again, participants were required to fixate throughout the target motion interval and their task was to estimate whether the target reappeared after occlusion at the right time, or too late.

The response screen was designed to be as similar as possible to the Visible Task. The words ‘ONTIME’ and ‘LATE’ were displayed on either side of the central fixation cross, with the position again indicating which hand corresponded to which response. Response hand was counterbalanced across conditions, and responses were unspeeded. After the response screen, there was 1.2 seconds of fixation before the static target appeared for the next trial. The task was split into 6 blocks. In each two-block chunk, every possible trial type occurred once (2 speed×2 direction x 2 reappearance error x 2 response hand x 5 occluder size). Half the participants did the blocks in the reverse order. The practice block again consisted of 16 trials with balanced stimulus parameters.

It is important to note that there were an equal number of on-time and late reappearance trials. This is different from the Visible Task, where velocity change oddballs were relatively infrequent. Nevertheless, both tasks were designed to encourage covert tracking while participants fixated.

### Analysis of Behavioral Data

Signal detection analysis was used to assess participants’ ability to identify oddballs in the Visible Task. In the Occluded Task, the proportion of trials judged to have reappeared on-time was analyzed as a function of Reappearance Error (on-time, late), Direction (left, right), and Speed (12, 20°/s) with a repeated measures ANOVA.

### EEG Analysis

Artifacts in the EEG data resulting from blinks, saccades or 50 Hz electrical noise were removed using Independent Components Analysis (ICA) [31]. Between 1 and 8 components were removed from each block (median = 4). The raw EEG was then segmented into epochs from −400 ms to 3092 ms around target onset. Epochs were baseline-corrected relative to a pre-target onset period of 200 ms. As stated above, oddball trials were excluded from analysis. The fact that oddballs occurred at unpredictable times prevented analysis of oddball-related ERPs.

Epochs with excessive ocular artifacts were excluded from analysis. This was identified by amplitudes exceeding 70 µv at electrodes AF7 or AF8, or by a correlation of >0.75 between AF7/AF8 and the horizontal EOG during the first 1700 ms. As mentioned above, two participants with <50% of trials remaining after this treatment were excluded from all further analysis. The number of trials included was reasonably high, and not significantly different between tasks (Visible Task, M = 86.51%, SD 12.52%, Occluded Task, M = 84.97%, SD 14.02%, *t* (17) = 0.475, *p* = 0.641).

To explore the patterns of ERP activity during the two tasks, sequences of topographic maps of scalp activity were produced [32], using grand average voltage at each electrode. Two clusters of electrodes were explored statistically. These were the right posterior electrodes (P2, P4, P6, P8, PO4, P08, O2, CP2, CP4 and CP6) and their left sided homologues (Figure 1C). Effects were explored with repeated measures ANOVAs. The Greenhouse-Geisser correction factor was applied when the assumption of sphericity was violated. Paired samples t tests were used to follow up significant interactions. Data points always comprised the average amplitude over a 40 ms window centered on the stated time point.

### Eye Position Analysis

While the eye position data from the eye tracker could have been used to exclude trials from the EEG analysis, the eye tracker may not detect various eye muscle artifacts because they do not produce large changes in eye position and apparent breaks of fixation can reflect temporary loss of signal from the eye tracker. It was therefore decided to use electrode-based exclusion criteria described above, but to use eye tracker data to corroborate that the vast majority (∼ 99%) of these trials did not include large eye movements at crucial intervals. Eye position data was also used to assess the relationship between eye position and target position. Trials where horizontal eye position deviated more than 2° from the median [33] were excluded from analysis of fixational eye movements (5.046%).

## Results

### Visible Task

Signal detection analysis revealed that all participants were sensitive to the velocity-change oddballs (average *d’* = 2.02, range 0.46 to 3.95), with d’ being significantly above chance (compared to zero using a one sample t test, *t* (17) = 8.857, *p*<0.001). All but one participant responded cautiously, with a bias towards reporting ‘no oddball’, with *c* being significantly greater than zero, that is, significantly different from the zero bias point (M = 0.66, *t* (17) = 7.655, *p*<0.001). The latter finding is important because it means that the ERPs were unlikely to be generated by the erroneous perception of oddballs in the normal trials.

**Figure 2:**
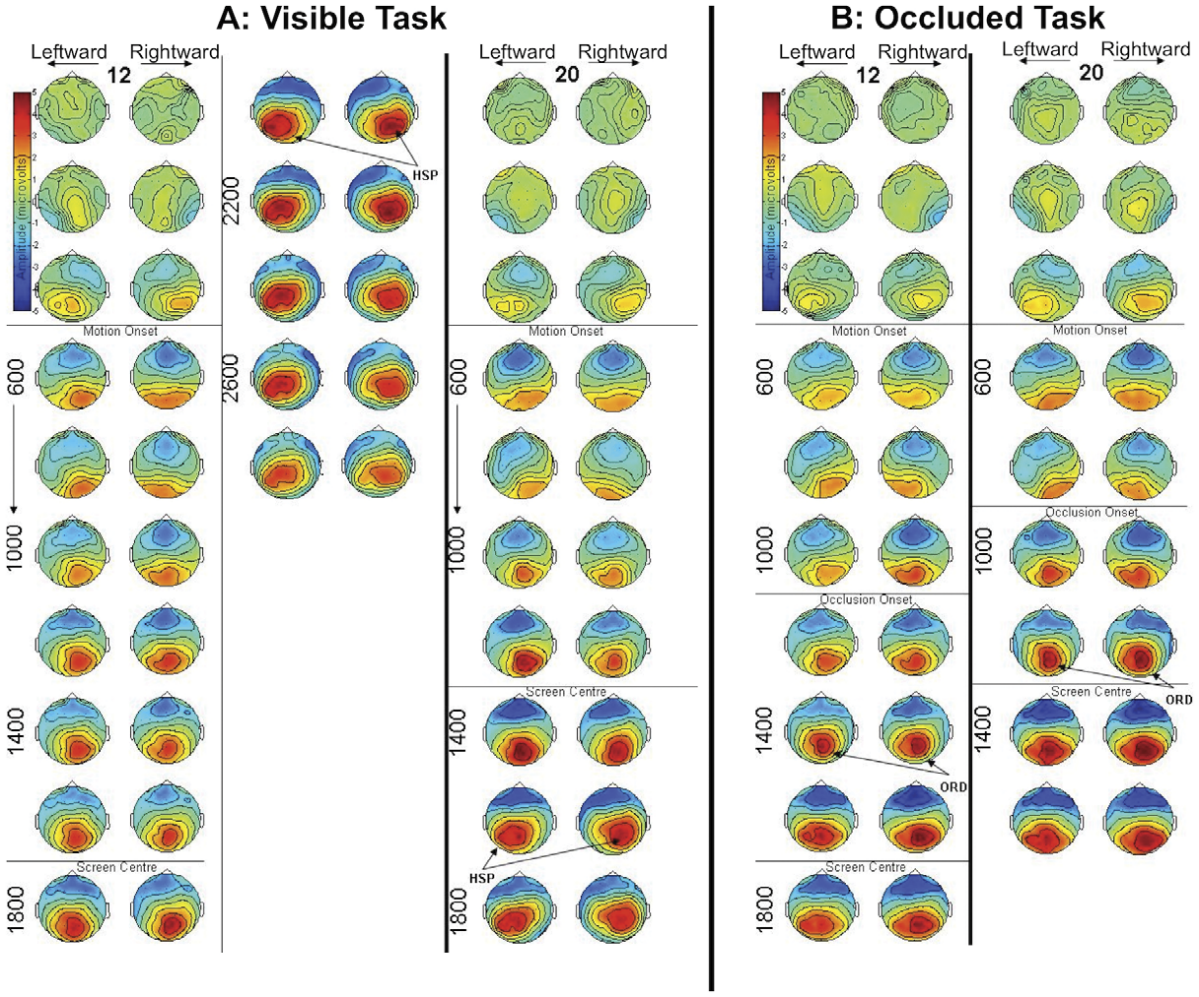
Sequential Topographies. A) Sequential topographies from the Visible Task. **B)** Sequential topographies from the Occluded Task. In A and B, leftward and rightward motion conditions are placed adjacently. For the Visible task, the 12°/s condition continues in another column. Each row represents a 200 ms interval. Maps show average amplitude over a 40 ms window around the stated time point. HSP  =  Hemifield Switch Positivity, ORD  =  Occlusion Related Deflection.

Sequences of grand-average topographic maps were produced in order to visualize ERP patterns in the Visible Task. Topographic plots were taken at 200 ms intervals in all four conditions (Speed: 12, 20°/s×Direction: leftward, rightward, Figure 2A). Oddball trials were excluded from all analyses.

Several patterns of activity were found in the data. These can be seen in Figure 2A. First, a posterior, positive potential developed in all conditions. This component was lateralized according to the direction of target motion. When the target moved leftward, the posterior positivity moved from right to left. Conversely, when the target moved rightwards, it moved in the opposite direction, from left to right. In both motion direction conditions, amplitude increased after the target passed the centre of the screen, and therefore this occurred later in the slow trials than the fast trials. The component also became more anterior towards the end of the trial.

**Figure 3:**
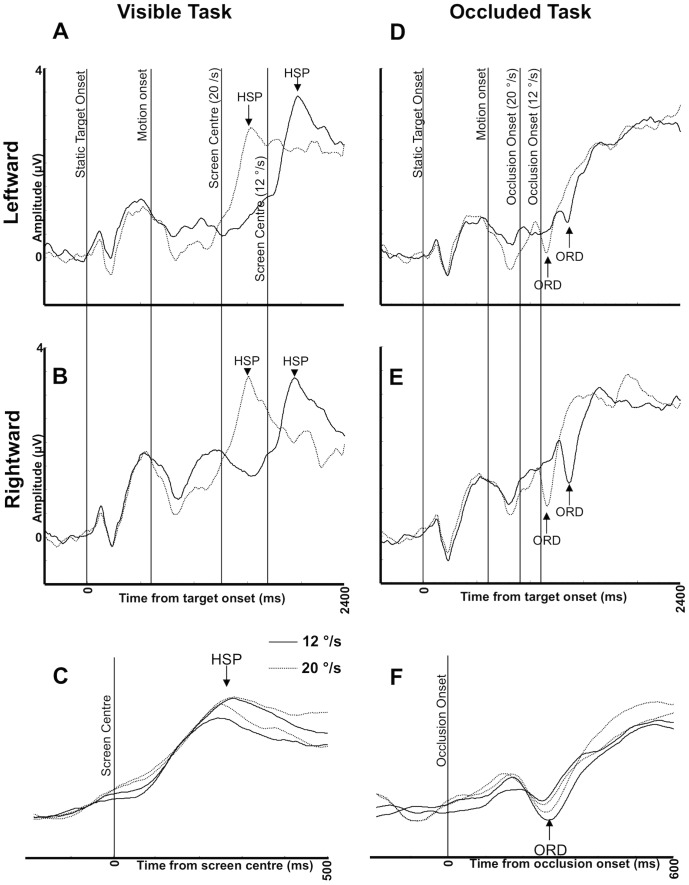
Event Related Potentials (ERPs). A) ERPs in the left cluster, leftward motion conditions of the Visible Task. B) ERPs from the right cluster, rightward motion conditions of the Visible Task. C) The Hemifield Switch Positivity (HSP) component in the Visible Task. Waveforms are realigned to the time the target passed the fixation cross, and baseline corrected to a 200 ms interval before this. D) ERPs in the left cluster, leftward motion conditions of the Occluded Task E) ERPs in the right cluster, rightward motion conditions of the Occluded Task. F) The Occlusion Related Deflection (ORD) component in the Occluded task. Waveforms are realigned to occlusion onset and baseline corrected to a 200 ms pre -occlusion interval. All ERP plots are smoothened with a 20 Hz filter and vertical lines illustrate events during the trial.

The posterior positivity shifted from central posterior electrodes to lateralized electrodes at around 200 ms after the targets crossed fixation. ERP topography at other parts of the trial was not so closely related to target location. This ERP may be described as the *Hemifield Switch Positivity* (HSP). These patterns are evident in the ERP plots of Figure 3A and B. Amplitude in the left electrode cluster is shown for the *leftward* motion condition, while amplitude in the *right* electrode cluster is shown for the *rightward* motion condition. It can be seen that after the target passed screen centre, there was a clear HSP in all conditions. This peaked around 240–260 ms after the target passed fixation. The latency of the HSP is exemplified in Figure 3C, which is realigned and baseline-corrected to the period 200 ms before the target reached fixation.

The patterns shown in Figure 3A, B and C were confirmed statistically. First we compared amplitude in the left and right posterior electrode clusters at the time of the HSP peak with a three factor repeated measures ANOVA [Side (left, right)×Direction (leftwards, rightwards)×Speed (12, 20°/s)]. The only significant effect was a strong Side×Direction interaction (*F* (1, 17) = 34.349, *p*<0.001). In the right cluster, amplitude was higher when the target moved rightwards (*t* (17) = 3.216; = 0.005), but in the left cluster HSP peak was greater when the target moved leftwards (*t* (17) = 5.527, *p*<0.001).

The latency of the HSP was explored by measuring the time point when amplitude was at 50% of the maximum (a standard measure of latency, [34]). Measurements were obtained from all but two participants, who were excluded because they did not show an HSP in every condition. Data from the remaining 16 participants was explored with a two factor repeated measures ANOVA [Direction (leftward, rightward)×Speed (12°/s 20°/s)], which revealed a main effect of Speed (*F* (1, 15) = 795.090, *p*<0.001, Figure 3A and B). There was no main effect of Direction or Direction×Speed interaction (*F* (1, 15) <1, NS). Next, the same data were standardized as a deviation from the time that the target passed fixation. Analysis of this standardized data found no effect of speed (*F* (1, 15) <1, N.S.), and no other effects (*F* (1, 15) <1, N.S). This confirms that HSP was indeed time-locked to the point when target crossed fixation (Figure 3C).

### Occluded Task

The proportion of trials judged to have reappeared ‘on-time’ was significantly greater when this was the appropriate response (83% vs. 44%, *F* (1, 17) = 264.12, *p*<0.001). This confirms that participants were covertly tracking the occluded targets.

Again, sequential topographic maps were aligned according to time from motion onset (Figure 2B). As for the Visible Task, posterior positive ERPs were lateralized according to motion direction, but the timing of the ERPs was different. In the Occluded Task, the positivity became focused on central electrodes around 200 ms post occlusion in all conditions. This was followed by a lateralized positive component, emerging ∼260 ms post occlusion. We refer to this ERP as the Occlusion Related Deflection (ORD). The latency of the ORD was not modulated by speed. In this experiment, the occlusion period always began before the target reached the fixation cross at the centre of the screen (Figure 1A). This meant that the lateralization of the posterior positivity occurred earlier than the HSP, particularly in the slower 12°/s condition (compare the Visible Task in Figure 2A with the Occluded Task in Figure 2B).

The latency of the ORD is demonstrated in Figure 3D and E. These plots depict ERPs in the left cluster for leftward motion conditions, and ERPs in the right cluster for rightward motion conditions. Until occlusion, these ERP waveforms were like those of the Visible Task. However, there was a small negative deflection at ∼180 ms post occlusion, and then a large positive deflection at ∼260 ms post occlusion. ORD latency was very similar in all motion conditions, as can be seen in Figure 3F, where the ORDs are aligned and baseline corrected to a 200 ms pre-occlusion period. The patterns shown in Figure 3 (D-F) were explored statistically. The ORD had a clear onset, but no clear peak. Therefore, the time point 100 ms after minimum occlusion duration was used for analysis. At this time, ERPs are likely to reflect occluded target tracking, and the positive component of the ORD was at, or near, maximum. Amplitude was analyzed as a function of Speed (12, 20°/s), Cluster (left, right) and Direction (leftward, rightward) with repeated measures ANOVA. There was a 3-way interaction (*F* (1, 17) = 5.246, *p* = 0.035), so we analyzed the 12 and 20°/s conditions separately. In the 12°/s condition, the only significant effect was a Cluster×Direction interaction (*F* (1, 17) = 10.987, *p* = 0.004). In the right cluster, amplitude was greater when the target moved rightwards (*t* (17) = −2.778, *p* = 0.013), while in the left cluster, amplitude was greater when the target moved leftwards (*t* (17) = 2.538, *p* = 0.021). In the 20°/s condition, results were less clear because the lateralization had not emerged by the end of the minimum occlusion duration. There was no Cluster×Direction interaction (*F* (1, 17) <1, NS). We acknowledge that this analysis of the 20°/s condition alone does not, in itself, support our conclusions regarding lateralization. Nevertheless, by comparing topographic development in the leftward and rightward trials of the occluded task, there is little doubt that the posterior positivity shifts with target motion, as it did in the visible task (Figure 2, right panels).

Next we explored latency by measuring the time at which the positive component of the ORD reached 50% of peak amplitude for each participant. Two participants did not show clear ORDs in all conditions, and were excluded (not the same two participants who were without the HSP, 16 participants remaining). Data were then analyzed as a function of Direction (left, right) and Speed (12°/s, 20°/s) with repeated measures ANOVA. As expected, 50% of peak amplitude occurred earlier in the 20°/s condition because the target reached the occluder earlier (*F* (1, 15) = 218.102, *p*<0.001, Figure 3D and E). There was no effect of Direction and no interaction (*F* (1, 15) <1, N.S.). Next, 50% peak time was measured as a deviation from occlusion onset, and then reanalyzed as above. This procedure removed the effect of Speed (*F* (1, 15) <1, N.S.), confirming that occlusion related components occurred at a fixed time after occlusion onset in all conditions (Figure 3F).

### Fixation Quality

Participants were required to fixate throughout all trials and track the moving targets covertly. Many blinks and large eye movement artifacts were removed from the raw EEG data with ICA, and remaining trials with activity indicative of eye movements were excluded. These methods removed high voltage electrophysiological artifacts produced by oculomotor muscles or movement of the retinal dipole. However, ICA does not eliminate *cortical activity* resulting from the visual effects of eye movements. Therefore, as with all EEG experiments, it remains possible that unwanted eye movements could have contributed to the ERPs [34].

To address this problem, EEG data were compared with eye position data acquired from the eye tracker. In general participants fixated well, however, there were a small number of trials where fixation was broken, but were nevertheless included in the EEG analysis. The frequency of eye movements around the time of relevant ERPs was therefore investigated. Eye movements were identified by eye position deviating by more than 2° from the median eye position. Samples where the eye tracker signal was lost were also conservatively defined as breaks of fixation. Eye movements during the 300 ms before HSP and ORD peaks were of particular interest. These intervals comprised 16 eye position data points.

Consider that a participant’s ERP was produced by averaging data across all trials included from a particular condition. In the Visible Task, there were thus 72 ERPs in total (18 participants×4 conditions). Importantly, 49% of ERPs did not include any trials contaminated by an eye movement and for those that did, only a small proportion of contributing trials were affected. For the worst participant, time point and condition, an eye movement in occurred in 8.8% of the trials. The mean value was 0.99%. Moreover, there was no positive correlation between participants’ contamination level and their ERP amplitude (Spearman’s Rho Coefficient <0.035, *p*>0.446, one tailed). This suggests that ERPs in the Visible Task did not reflect unwanted eye movements.

In the Occluded Task, 68% of ERPs were totally uncontaminated. The maximum proportion of trials contaminated was 8.82% (M = 0.21%). Again, there was no relationship between contamination and ERP amplitude (Spearman’s Rho Coefficient <0.247, *p*>0.162, one-tailed). This suggests that large eye movements did not cause the ERPs in the Occluded Task.

### Fixational Eye Movements

**Figure 4:**
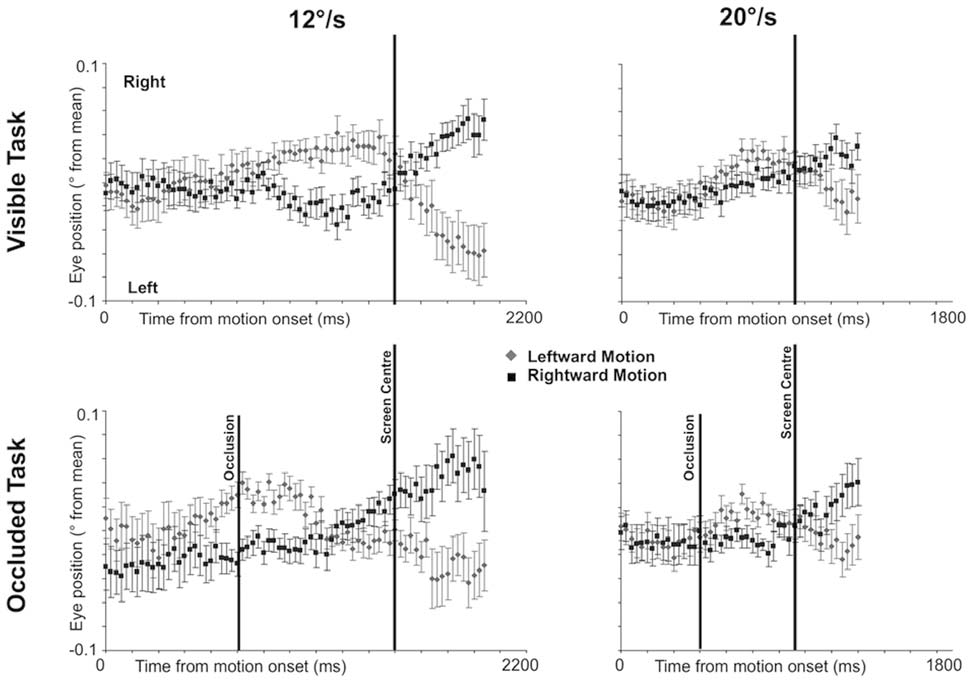
Fixational eye movements. Standardized eye position is shown as a function of time since target onset in the 12 and 20°/s conditions of the Visible and Occluded Tasks. Error Bars  =  +/−1 S.E.M.

Next we analyzed small changes in grand average eye position. For this analysis, all trials where fixation was broken were excluded. Each participant’s eye position data was averaged across all valid trials and then standardized against their mean value [25]. Figure 4 shows changes in standardized eye position in both tasks. Note, however, that the spatial precision of the eye tracker (∼0.5°) meant we could not detect individual microsaccades. In the Visible Task, it can be seen that mean eye position shifts around the time that the target reached fixation. The direction of the shift is dictated by motion direction: eye position shifted leftwards when the target moved to the left and it shifted rightwards when the target moved to the right. This happened later in the 12°/s condition than the 20°/s condition. In the Occluded Task, patterns were similar. However, this shift seemed more related to occlusion onset, and was not so tightly related to the target moving across the fixation point.

Separate Direction×Time repeated measures ANOVAs were used to investigate patterns in each panel of Figure 4. These ANOVAs explored time points sampled every 100 ms, rather than every available time point. This was because inclusion of too many levels prevents analysis of sphericity, and can also lead to interactions that do not reflect robust patterns in the data. The time points used in a particular ANOVA were chosen to capture the cross-over effect between leftward and rightward motion conditions. The patterns seen in Figure 4 were confirmed by Direction×Time interactions in all analyses (*F* (1.749, 29.733) >4.045, *p*<0.034).

## Discussion

In the Visible Task, participants observed a single moving dot while fixating. They were able to detect rare velocity-change oddballs, confirming that they were covertly tracking the visible moving targets. In the Occluded Task, the target travelled behind an invisible occluder, and participants were able to discriminate whether the target reappeared on-time or 300 ms too late, implying that they were also covertly tracking the occluded moving targets [8], [35].

The current work explored two unanswered questions about the neural correlates of covert tracking. First, previous literature has provided a mixed account of whether the right hemisphere is more important than the left in covert tracking. The right hemisphere is known to be more involved in visuospatial operations such as mental object rotation [36], and right hemisphere damage produces more profound deficits in spatial orienting than equivalent left sided lesions [14]. Meanwhile, fMRI data suggest that the frontoparietal attention network is generally bilaterally activated when people track a single moving target [17]. Finally, in our earlier ERP study [21], we found right lateralized ERPs during visible and occluded tracking, which could have been due to hemispheric asymmetry, but could have been due to the fact that we presented rightward motion only.

The current experiment demonstrated conclusively that ERPs related to covert tracking are not invariably right-sided. Both tasks produced a comparable positive potential at posterior electrode sites. This component shifted across the scalp depending on the direction of the moving target. When the target moved rightwards, the positivity shifted from the left to right hemisphere electrode sites. Conversely, when the target moved leftwards, the positivity moved leftwards. The component always reached peak amplitude in the second half of the trial. In the Visible Task, we refer to this ERP as the Hemifield Switch Positivity (HSP). In the Occluded Task, the same pattern was found (although there was additional effect attributable to occlusion onset, termed the Occlusion Related Deflection, ORD). In other words, the development of ERPs in the rightward motion condition was a mirror image of ERP development in the leftward motion condition. We conclude that covert tracking of visible and occluded targets are not exclusively right hemisphere functions, but rather that the involvement of left or right hemisphere modules depends on motion direction.

Other work has also explored ERPs generated by covert shifts of visuospatial attention. For example, the Anterior Directing Attention Negativity (ADAN) and Late Directing Attention Positivity (LDAP) components are both lateralized according to the direction of large, covert shifts of attention [23], [37]. The characteristics of the ERPs found in the present study may also be related to those found by Praamstra et al. [32], who also recorded a positive ERP component ipsilateral to the direction of covert attention. However, the current study differs from those above by exploring continuous tracking, rather than single abrupt shifts in spatial attention.

We anticipated two ways in which ERPs might differ between Visible and Occluded tasks: First that occluded tracking would involve more right hemisphere regions, as some studies showing additional right sided during occlusion [17], [20]. However, we did not find exclusively right lateralized ERP development during the Occluded Task. Instead, like the Visible Task, the development of ERPs in the leftward motion condition was approximately a mirror image of ERPs in the rightward motion condition, suggesting that both hemispheres contribute equally. Second, we predicted that there would be a change in brain activity at 200 ms post occlusion in the Occluded Task. This was found: the ORD occurred at the predicted time point.

There is now strong evidence that memory-guided tracking begins at around 200 ms post occlusion [11], [12], and that memory guided tracking involves activation of the Dorsolateral Prefrontal Cortex (DLPFC, [17], [38], [39]). More specifically, it is possible that the top-down inputs from DLPFC maintain activity in the network of frontal and parietal regions responsible for spatial attention, when visual velocity signals become unavailable. Rather than generating the ORD directly, we suggest that it is likely that inputs from the DLPFC indirectly contributed to ERPs measured over the parietal cortex. At any rate, it can be seen that the timing of the ORD was consistent with existing accounts of the onset of memory-guided tracking [21].

The present study also tested the hypothesis that components of the oculomotor system mediate covert tracking. Perhaps the ideal test would have been to measure ERPs during separate covert tracking and smooth pursuit conditions, however, the artifacts from the ocular muscles precluded this approach. Instead, we examined the hypothesis by recording small changes in eye position that always occur during fixation. We found that, even on trials where participants successfully maintained fixation, average eye position moved slightly leftwards when the target moved to the left, and slightly rightwards when the target moved to the right. It is likely that these fixational eye movements were produced by dynamic conflict between the oculomotor control system (which, we hypothesize, was engaging with the moving targets) and fixation commands (which were blocking the execution of large eye movements) [27], [28]. The pattern of fixational eye movements recorded here builds on the results of Makin and Poliakoff [25], by demonstrating that changes in eye position around fixation are dependent on target direction.

Interestingly, comparison of the timing of the fixational eye movements and ERPs suggests that both were produced by the same processes: in the Visible Task, the largest shift in eye position was approximately time-locked to the point when the target reached the centre of the screen, just like the HSP. In the Occluded Task, the shift in mean eye position occurred around 200 ms after occlusion onset, just like the ORD. ERPs and fixational eye movements could therefore be independent reflections of the underlying covert tracking mechanisms, since both metrics were related to the stimuli in the same way. In fact, given this close relationship, one might argue that that the fixational eye movements caused the ERPs directly. Indeed, Dimigen et al. [40] found that small eye movements (known as microsaccades) produce a cortically generated, positive, ERPs ipsilateral to the direction of the eye movement, reflecting shifts in the visual field produced by the microsaccade. It is impossible to ascertain the role of microsaccades in our experiment with confidence, as the eye tracker lacked the resolution to identify individual microsaccades. However, if fixational eye movements caused the ERPs, then participants who show a clear shift in mean eye position would have larger ERPs and vice versa. When we tested this hypothesis, we found no positive correlation between magnitude of the fixational eye movement effect and ERP amplitude in any condition (Spearman’s Rho Coefficient <0.35, *p*>0.084, one tailed). This suggests that fixational eye movements did not cause the ERPs. However, the fact the amplitude of these metrics did not correlate at a between participants level does not mean that they cannot reflect the same cognitive events, since individual differences in fixational eye movements are likely to reflect differences in inhibitory ability, rather than the degree of oculomotor activation produced, while ERP magnitude could reflect idiosyncrasies in cortical folding.

The interpretation of our fixational eye movement data is consistent with the pre-motor theory of attention [22], [24], which has argued that discrete shifts of spatial attention are produced by pre-motor systems responsible for saccadic eye movements, even if eye movements themselves are never executed [41]. Our results are also in agreement with previous neuroimaging studies that suggest a common network of brain regions controls covert tracking and smooth pursuit [15], [17], [18]. It is also worth considering that the separation of spatial attention and pursuit is not typical in real world situations, and during smooth pursuit, attention is focused on the pursuit target rather than other regions of space [42]. Meanwhile, after the initial *open loop phase,* (where pursuit is a reflexive response to summed motion in the scene), spatial attention is used to select pursuit targets [43], [44]. Given this additional evidence, it is reasonable to suggest that some modules of the pursuit pathway were active during our task, and that this led to the fixational eye movement profile we recorded.

Attention to space and attention to moments in time have been considered to be partially dissociable systems, although they can serve the same goals – namely enhancing task relevant inputs [3]. In the case of covert tracking, spatial attention works in very close harmony with temporal attention: the attentional spotlight must be continually shifted to exactly the right place at exactly the right time, to keep up with the target [8], [10], [45]. The smooth pursuit eye movement system is perfectly adapted for such spatiotemporal integration. During smooth pursuit, velocity information is extracted from retinal motion signals, and combined with top-down, predictive velocity signals in order to keep the fovea aligned with the moving target [5], [46]. We think it is likely that covert pursuit makes use of this pre-existing velocity processing network, even if eye movements are inhibited at a later stage.

### Conclusions

This study investigated the mechanisms that control covert attentive tracking of visible and occluded targets. We found that ERPs related to the direction of target motion were symmetrical; that is, the contribution of each hemisphere depends on the horizontal direction of target motion. We also found small shifts in eye position lateralized according to the direction of target motion, implying covert activation of the oculomotor system.

We finish by putting this work into context: after all, a great deal is known about the cognitive and neural basis of visuospatial attention. Most research has investigated abrupt shifts of attention to discrete spatial locations, or, more recently, on attention to objects that appear at a predictable point in time [3]. The frontoparietal network controls spatial attention, with the right hemisphere possibly playing a dominant role [13]. Meanwhile, the pre-motor theory of attention emphasizes the overlap between covert attentive shifts and saccadic eye movements [24]. The current work studied the simultaneous control of spatial and temporal attention during covert attentive tracking. The results were compatible with the pre-motor theory, but they highlight the overlap between covert tracking and smooth pursuit, rather than the better-known link between attentive shifts and saccades. We also conclude that covert tracking is *not* an exclusively right hemisphere operation: instead the balance of left and right activity depends on the current location of the moving target.
